# Dental Societies in America

**Published:** 1887-02

**Authors:** 


DENTAL SOCIETIES IN AMERICA.

If we were to gauge the fraternal feeling among dentists of different nationalities by the number of dental societies and the frequency of dental meetings, America would be far in advance. There are probably more dental associations in and immediately about New York City than exist in any other nation of the world. There is an intense thirst here, both to obtain and to give information on professional subjects, and it is to this fact that American dentistry owes its high standing. When a practitioner of any reputation or professional standing here has made an advance or devised a new method or implement, he knows no rest until he has made his brethren acquainted with it. Our journals are continually filled with descriptions of new appliances and modes of practice, and in this they are in advance of those of any other people. There is so much of this intercommunication of ideas, that journals are continually multiplied. There are but three exclusively dental journals published in Great Britain. There are over twenty in America. There are eight (we think) dental societies in England. Caulk's Annual enumerates over one hundred in the United States.

Nor is it in number of societies alone that our sodality is peculiar. Our meetings are of an eminently practical character. English meetings are more decorously conducted, English dentists look more carefully after ethical matters, and at their meetings discuss questions of a broader scientific character, but our clinics and practical demonstrations are almost unknown there, and, in fact, are considered rather *infra. dig.* As a consequence, English dentists know more of general science than their American brethren, but less of practical dentistry.

